# The relationship between mental toughness, stress, and mindfulness in climbers

**DOI:** 10.3389/fpsyg.2026.1799925

**Published:** 2026-08-14

**Authors:** Hulusi Alp, Reha Bozgüney, İbrahim Kubilay Türkay, Görkem Turaç, Zeynep Senem Söyleyici Öcal, Didem Yazar, Eyüp Temur, Banu Can

**Affiliations:** 1 Department of Recreation, Faculty of Sport Sciences, Suleyman Demirel Universitesi, Isparta, Türkiye; 2 Department of Recreation, Faculty of Sport Sciences, Istanbul Galata Universitesi, Beyoğlu, Türkiye; 3 Department of Recreation, Faculty of Sport Sciences, Selcuk Universitesi, Konya, Türkiye; 4 Department of Sport Management, Faculty of Sport Sciences, Ardahan Universitesi, Ardahan, Türkiye

**Keywords:** climbing, mental toughness, mindfulness, sport psychology, stress

## Abstract

**Background:**

Climbing is a sport that demands high levels of both physical and mental effort, and athletes may encounter significant stress during performance. Mental toughness refers to an athlete’s capacity to cope with stress and maintain performance under challenging conditions. Mindfulness is the conscious awareness and experiential engagement with the present moment. This study aimed to examine the relationship between mental toughness and stress levels in climbers, and to investigate the moderating role of mindfulness in this relationship.

**Methods:**

The study included 150 active climbers (90 males, 60 females; mean age = 25.4 ± 4.7 years). Mental toughness was assessed using the Sports Mental Toughness Questionnaire (SMTQ) and the Mental Toughness Inventory (MTI), stress levels were measured via the Perceived Stress Scale (PSS-10), and mindfulness was evaluated using the Mindful Attention Awareness Scale (MAAS). Multiple regression and moderation analyses were conducted using SPSS and the PROCESS macro, with 5,000 bootstrap samples and 95% confidence intervals.

**Results:**

Correlation analyses indicated significant negative relationships between SMTQ and MTI scores and PSS-10 scores, as well as between MAAS scores and PSS-10 scores (*p* < 0.001). Multiple regression analyses revealed that SMTQ (*β* = −0.33), MTI (*β* = −0.38), and MAAS (*β* = −0.29) significantly and negatively predicted stress levels (*p* < 0.001). Moderation analysis showed that higher levels of mindfulness strengthened the negative effect of mental toughness on stress (Interaction *β* = −0.18, *p* = 0.002).

**Conclusion:**

These findings indicate that both mental toughness and mindfulness play a crucial role in stress management among climbers. In particular, mindfulness enhances the stress-reducing effects of mental toughness. The results provide practical implications for sport psychology interventions and climbing training programs.

## Introduction

Climbing is a sport that demands high levels of both physical and mental effort, precision, and concentration. Climbers often face unique psychological challenges due to the high-risk nature of the sport and the need for sustained focus on technically demanding routes (MacKenzie and Jones, 2020; Mellalieu et al., 2009). Such conditions can elevate perceived stress, which may negatively impact performance, motivation, attention, and overall psychological well-being (Fletcher and Sarkar, 2012; Gucciardi and Gordon, 2011; Jones et al., 2007). Therefore, effective stress management is critical for climbers aiming to achieve consistent high-level performance (Hanton et al., 2005; Nicholls et al., 2009).

Mental toughness, defined as an athlete’s ability to maintain focus, cope effectively with stress, and sustain performance under pressure, has been identified as a key psychological resource for high-performance athletes (Clough and Strycharczyk, 2012; Gucciardi et al., 2017). In climbing, mental toughness enables athletes to manage fear, maintain concentration on complex sequences, and persevere through physically and mentally demanding climbs (Cowden, 2017; Filimon et al., 2021). Empirical evidence indicates that athletes with higher mental toughness experience lower levels of perceived stress and demonstrate greater resilience in challenging competitive and training environments (Turkington et al., 2023a; Mojtahedi S. et al., 2021).

Mindfulness, characterized as non-judgmental awareness of the present moment (Kabat-Zinn, 2003), has emerged as a valuable psychological skill in sport contexts. Mindfulness-based practices have been shown to improve emotional regulation, attentional focus, and cognitive flexibility, all of which support performance under pressure (Gardner and Moore, 2007; Kaufman et al., 2009; Baltzell and Akhtar, 2014; Noetel et al., 2019). In climbing, mindfulness may help athletes maintain situational awareness, manage fear of heights, and respond adaptively to unexpected challenges on the wall (Sappington and Longshore, 2015; Rogowska, 2024). Moreover, mindfulness can enhance the stress-buffering effects of mental toughness by enabling climbers to apply their resilience skills more effectively when facing high-pressure situations (Sappington and Longshore, 2015; Birrer et al., 2012).

Taken together, these findings suggest that both mental toughness and mindfulness are critical for effective stress management in climbers. However, the interactive effects of these psychological constructs remain underexplored. Specifically, it is unclear whether mindfulness moderates the relationship between mental toughness and perceived stress in climbing contexts. Understanding this interaction is particularly important for designing evidence-based interventions aimed at enhancing climbers’ psychological resilience and performance.

### Based on this theoretical and empirical rationale, the following hypotheses were formulated

*H1*: higher levels of mental toughness are associated with lower perceived stress.

*H2*: higher levels of mindfulness are associated with lower perceived stress.

*H3*: mindfulness moderates the relationship between mental toughness and perceived stress, such that the negative association between mental toughness and stress becomes stronger at higher levels of mindfulness.

From a theoretical perspective, mindfulness is conceptualized as a regulatory (moderating) mechanism rather than a mediating variable in the relationship between mental toughness and perceived stress. While mental toughness represents a relatively stable psychological capacity that enables individuals to cope with pressure, mindfulness reflects a context-sensitive attentional and emotional regulation process that can influence how effectively these capacities are utilized under stress. According to the stress-buffering model, personal resources do not operate in isolation but interact with regulatory processes that shape stress perception. In this regard, mindfulness may alter the strength of the relationship between mental toughness and perceived stress by facilitating present-moment awareness and reducing cognitive reactivity. Therefore, rather than transmitting the effect of mental toughness (as in mediation), mindfulness is expected to condition the effectiveness of mental toughness in stress regulation, justifying its role as a moderator.

## Methods

### Study design and participants

His study was conducted using a cross-sectional correlational design to examine the relationships among mental toughness, mindfulness, and perceived stress in climbers, as well as the moderating role of mindfulness on the mental toughness–stress relationship. Data were collected at a single point in time, and the analyses focused on associations between variables without inferring causal relationships.”

Participants were recruited using a convenience sampling method through climbing gyms and sports clubs. Data were collected via an online questionnaire distributed through social media platforms and direct contact with climbing communities. Participation was voluntary, and all participants provided informed consent prior to data collection.

A total of 150 sport climbers participated in the study. The sample size was determined using G*Power 3.1, assuming 80% power, *α* = 0.05, and a medium effect size (*f*^2^ = 0.15) for moderation analysis. The analysis indicated that a minimum of 107 participants was required; therefore, a sample of 150 participants was considered sufficient (Table 1). The mean age of participants was 25.4 ± 4.7 years (range: 18–35), with an average climbing experience of 4.8 ± 2.9 years (range: 1–12) and weekly training duration of 6.2 ± 2.1 h. The sample included 90 males (60%) and 60 females (40%).

**Table 1:** Participant characteristics.

Characteristic	*n*	(%)	Mean	SD	Description
Total participants	150	100%			Active sport climbers
Male	90	60%			Gender distribution
Female	60	40%			Gender distribution
Age (years)			25.4	4.7	Mean ± SD (min–max: 18–35)
Climbing experience (years)			4.8	2.9	Mean ± SD (min–max: 1–12)
Weekly training duration (hours)			6.2	2.1	Average training hours

Inclusion criteria were:

- Active participation in climbing.
- Age between 18 and 35 years.
- At least 1 year of regular climbing experience.

Exclusion criteria were:

- Serious injury within the last 6 months.
- Current psychiatric treatment.

For moderation and mediation analyses, 5,000 bootstrap samples were used. Bootstrap sampling was selected as it provides reliable confidence intervals and non-parametric estimates even when data distribution assumptions are not met (Preacher and Hayes, 2008). The use of 5,000 bootstrap samples is a widely accepted standard to ensure robust estimates and sufficient statistical power.

### Measure

Four instruments were used to assess mental toughness, perceived stress, and mindfulness among climbers.

### Mental toughness

Two scales were employed: the Sports Mental Toughness Questionnaire (SMTQ) and the Mental Toughness Inventory (MTI). The SMTQ (Sheard et al., 2009). Includes 14 items across three subscales: confidence, control (emotional and life control), and constancy (stability under pressure), with Cronbach’s *α* = 0.81. The MTI (Gucciardi et al., 2015) consists of 15 items measuring cognitive flexibility, emotional control, and constancy, with reliability coefficients ranging from 0.82 to 0.94.

### Perceived stress

The 10-item Perceived Stress Scale (PSS-10; (Cohen et al., 1983)) was used to assess participants’ perceived stress over the past month. Cronbach’s *α* in the present sample was 0.87.

### Mindfulness

Mindfulness was measured using the Mindful Attention Awareness Scale (MAAS; (Brown and Ryan, 2003), a 15-item instrument evaluating present-moment awareness in daily life. Cronbach’s α in the present study was 0.91.

### Demographics

Participants’ age, gender, climbing experience, and weekly training hours were collected via a structured questionnaire. These variables were used to describe the sample and included as control variables in subsequent analyses.

### Data analysis

Prior to statistical analyses, data were screened for outliers, missing values, and normality assumptions. Missing data were below 5% and were handled using mean imputation. Outliers were assessed via Mahalanobis distance, and two participants exceeding the threshold were removed. Normality was evaluated through skewness and kurtosis values, all within ±1.5, indicating approximate normal distribution.

Regression assumptions were also tested. Linearity was examined through scatterplots, homoscedasticity was assessed via residual plots, and independence of errors was confirmed using the Durbin–Watson statistic (DW = 1.89). No violations were detected.

Pearson correlation analyses were conducted to examine relationships among study variables. Multicollinearity was assessed using Variance Inflation Factor (VIF) and tolerance values. All independent variables had VIF < 2 and tolerance >0.50, indicating no multicollinearity issues (Table 2).

**Table 2:** Multicollinearity analysis of independent variables.

Variable	Tolerance	VIF
SMTQ	0.72	1.38
MTI	0.69	1.45
MAAS	0.75	1.33

Additionally, although mental toughness and mindfulness were moderately correlated, multicollinearity diagnostics (VIF < 2) indicated that these constructs represent statistically distinct variables. This supports their conceptual differentiation and justifies their inclusion in the same regression model.

Multiple linear regression analyses (enter method) were conducted to test Hypotheses 1 and 2, with perceived stress (PSS-10) as the dependent variable, and mental toughness (SMTQ, MTI) and mindfulness (MAAS) as independent variables. Model significance was assessed via F-test, and the contribution of each predictor was evaluated using *β* coefficients, t-values, and 95% confidence intervals. Results indicated significant negative effects of SMTQ (*β* = −0.33, *p* < 0.001), MTI (*β* = −0.38, *p* < 0.001), and MAAS (*β* = −0.29, *p* < 0.001) on perceived stress, suggesting that higher mental toughness and mindfulness are associated with lower perceived stress.

To test Hypothesis 3, a moderation analysis was conducted to examine whether mindfulness (MAAS) moderates the relationship between mental toughness (MTI) and perceived stress (PSS-10). The PROCESS Macro v4.2 (Model 1; (Hayes, 2018)) was used. All variables were mean-centered, and an interaction term (MTI × MAAS) was created to test whether the effect of mental toughness on perceived stress depends on mindfulness levels. The interaction was significant (*β* = −0.18, *t* = −3.11, *p* = 0.002, 95% CI [−0.29, −0.07]), indicating that higher mindfulness strengthens the negative association between mental toughness and perceived stress.

All analyses were performed using IBM SPSS Statistics 29 and PROCESS Macro v4.2. Statistical significance was set at *p* < 0.05.

## Results

Table 3 presents the descriptive statistics of the psychological scales administered to the participants. The mean SMTQ score was 52.3 ± 7.8 (range: 34–70), the mean MTI score was 84.7 ± 12.1 (range: 58–110), the mean PSS-10 score was 18.5 ± 5.6 (range: 7–31), and the mean MAAS score was 64.2 ± 8.4 (range: 45–82).

**Table 3:** Descriptive statistics.

Scale	N	mean	SD	Min	Max
SMTQ	150	52.3	7.8	34	70
MTI	150	84.7	12.1	58	110
PSS-10	150	18.5	5.6	7	31
MAAS	150	64.2	8.4	45	82

Table 4 displays the correlation matrix of the psychological scales. SMTQ and MTI were positively and strongly correlated (*r* = 0.64, *p* < 0.001). Perceived stress (PSS-10) was negatively correlated with both SMTQ (*r* = −0.46, *p* < 0.001) and MTI (*r* = −0.52, *p* < 0.001). Mindfulness (MAAS) was positively correlated with SMTQ (*r* = 0.37, *p* < 0.001) and MTI (*r* = 0.42, *p* < 0.001), and negatively correlated with PSS-10 (*r* = −0.41, *p* < 0.001) (see Table 5).

**Table 4:** Correlation matrix.

Variable	1.	2.	3.	4.
1. SMTQ	–			
*p*				
*n*				
2. MTI	0.64	–		
*p*	0.001			
*n*	150			
3. PSS-10	−0.46	−0.52	–	
*p*	0.001	0.001		
*n*	150	150		
4. MAAS	0.37	0.42	−0.41	–
*p*	0.001	0.001	0.001	
*n*	150	150	150	

**Table 5:** Multiple regression analysis (H1, H2 and H3).

Dependent variable	Independent variable	Moderator	*β*	*t*	*p*	95% CI	*R* ^2^	F (df)
PSS-10	SMTQ	–	−0.33	−5.12	<0.001	[−0.44, −0.22]	0.45	39.77 (3,146)
	MTI	–	−0.38	−5.89	<0.001	[−0.50, −0.26]	0.45	39.77 (3,146)
	MAAS	–	−0.29	−4.75	<0.001	[−0.40, −0.17]	0.45	39.77 (3,146)
PSS-10	MTI	MAAS	−0.18	−3.11	0.002	[−0.29, −0.07]	0.48	45.12 (3,146)

This table presents the results of multiple regression analyses testing Hypotheses 1 and 2, as well as the moderation analysis for Hypothesis 3. Standardized regression coefficients (*β*), *t*-values, *p*-values, and 95% confidence intervals (CI) are reported for each predictor. *R*^2^ and *F* statistics indicate the proportion of variance explained by the model and overall model significance, respectively. The interaction term (MTI × MAAS) represents the moderating effect of mindfulness (MAAS) on the relationship between mental toughness (MTI) and perceived stress (PSS-10). Higher mindfulness strengthened the negative association between mental toughness and stress.

**Figure 1:**
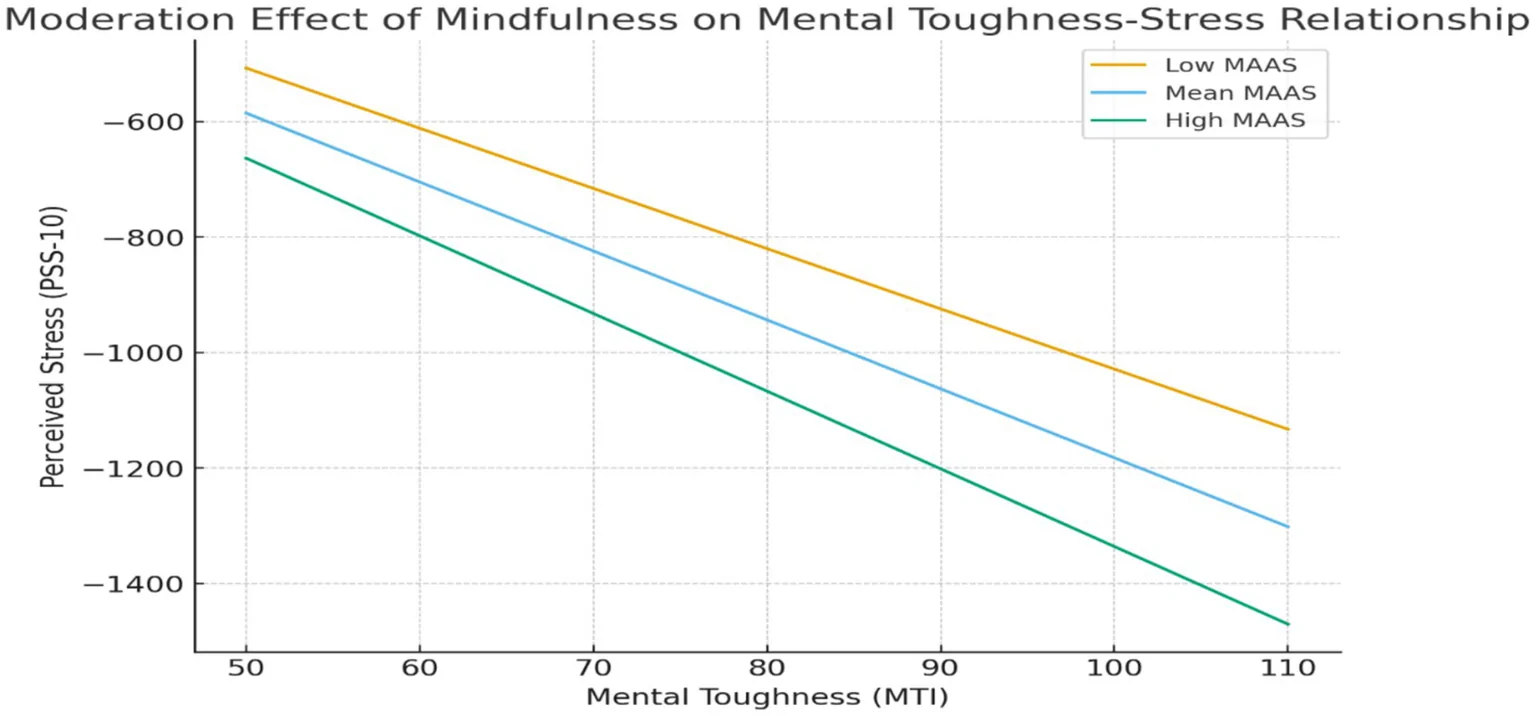
Moderating effect of mindfulness: higher mindfulness strengthens the negative association between mental toughness and perceived stress.

Figure 1 shows that climbers with higher mindfulness scores (green line) exhibit a stronger reduction in perceived stress as mental toughness increases, while climbers with lower mindfulness scores (orange line) show a weaker reduction.

## Discussion

The present study examined the associations between mental toughness (SMTQ, MTI), mindfulness (MAAS), and perceived stress (PSS-10), as well as whether mindfulness moderates the relationship between mental toughness and perceived stress. Findings revealed that all predictors—mental toughness indices and mindfulness—were significantly and negatively associated with perceived stress, and that mindfulness strengthened the negative effect of mental toughness on stress. These findings may provide theoretical and practical insights into how resilience mechanisms interact in stress regulation.

### Main findings and theoretical implications

Consistent with previous evidence, individuals with higher mental toughness reported lower perceived stress (Turkington et al., 2023b; Mojtahedi A. et al., 2021). Mental toughness enables composure, confidence, and cognitive control under pressure, allowing stressors to be perceived as manageable rather than threatening (Clough et al., 2016; Cowden and Meyer-Weitz, 2022). This aligns with resilience models emphasizing that personal hardiness buffers against psychological strain (Wister et al., 2022).

Similarly, mindfulness was negatively associated with perceived stress, in line with meta-analyses confirming that mindfulness-based interventions reliably reduce stress and enhance well-being (Pan et al., 2024; Sadooghiasl et al., 2022). Mindfulness fosters present-moment awareness and attentional control, reducing maladaptive cognitive reactivity (Keng et al., 2011; Creswell, 2017). Trait mindfulness also promotes emotional stability and adaptive coping across populations (Duggan et al., 2024; Chen, 2021).

### Moderating effect of mindfulness

The moderation analysis revealed a significant interaction between mental toughness and mindfulness (*β* = −0.18, *p* = 0.002). Figure 1 shows that climbers with higher mindfulness scores experience a stronger stress-reducing effect of mental toughness, while those with lower mindfulness scores show a weaker effect. This suggests that the combination of high mental toughness and high mindfulness is most beneficial for stress reduction. This supports dual-component resilience models, which emphasize the balance between stability (toughness) and flexibility (mindfulness) (Birrer et al., 2012; Wister et al., 2022). Mindfulness may enhance adaptive components of mental toughness—such as control, confidence, and perseverance—by fostering present-moment awareness and emotional regulation.

### Practical implications

These findings highlight the value of integrated interventions combining toughness training with mindfulness-based components. Mindfulness-based cognitive approaches (MBCT) or short MBSR modules can complement toughness-focused programs by fostering emotional flexibility alongside cognitive control (Pan et al., 2024; Oguntuase et al., 2022). Digital or mobile-based mindfulness programs may further enhance accessibility and adherence (Tanaka et al., 2023). Such integrated programs can benefit athletes, educators, and health professionals by strengthening stress tolerance (Rogowska, 2024; Chen, 2021).

### Limitations and future directions

This study used a cross-sectional design, which limits causal inferences between mental toughness, mindfulness, and perceived stress. Although the sample of 150 climbers provides initial insights, caution is needed when generalizing to other populations, sports, age groups, or cultural contexts. Future research should employ longitudinal or experimental designs to clarify causal relationships and examine whether the observed moderation effect holds across different sports disciplines, age ranges, and cultural backgrounds. Additionally, exploring these dynamics in non-athlete populations could broaden the applicability of the findings.

An alternative explanation worth considering is whether mindfulness could function as a mediator rather than a moderator. Although the present study focused on moderation based on theoretical grounds, future research could compare alternative models (e.g., mediation vs. moderation) using longitudinal or experimental designs to further clarify the underlying mechanisms.

## Conclusion

In summary, both mental toughness and mindfulness independently and are associated with lower perceived stress, with mindfulness enhancing the protective effect of mental toughness. These findings provide a theoretical foundation for designing evidence-based stress management programs that cultivate both stability and flexibility, promoting optimal psychological well-being in academic, clinical, and sport settings.

## Data Availability

The datasets presented in this study are not publicly available because they contain identifiable participant information. Requests to access the datasets should be directed to Reha Bozgüney (reha.bozguny@gmail.com).

## References

[ref1] Baltzell A. Akhtar V. (2014). Mindfulness meditation training for sport (MMTS) intervention: impact with intercollegiate athletes. J. Clin. Sport Psychol. 8, 285–307. doi: 10.1123/jcsp.2014-0013

[ref2] Birrer D. Röthlin P. Morgan G. (2012). Mindfulness to enhance athletic performance: theoretical considerations and possible impact mechanisms. Mindfulness 3, 235–246. doi: 10.1007/s12671-012-0109-24

[ref3] Brown K. W. Ryan R. M. (2003). The benefits of being present: mindfulness and its role in psychological well-being. J. Pers. Soc. Psychol. 84, 822–848. doi: 10.1037/0022-3514.84.4.822, 12703651

[ref4] Chen L. (2021). Mindfulness, stress, and well-being: a systematic review in sport and exercise contexts. Int. J. Sport Psychol. 52, 355–374. doi: 10.7352/ijsp.2021.052

[ref5] Clough P. Earle K. Sewell D. (2016). “Mental toughness: the concept and its measurement,” in Solutions in sport Psychology, ed. Cockerill I. (Stamford: Thomson Learning), 32–45.

[ref6] Clough P. Strycharczyk D. (2012). Developing mental Toughness: Improving Performance, Wellbeing and Positive Behaviour in others. London: Kogan Page.

[ref7] Cohen S. Kamarck T. Mermelstein R. (1983). A global measure of perceived stress. J. Health Soc. Behav. 24, 385–396. doi: 10.2307/2136404, 6668417

[ref8] Cowden R. G. (2017). Mental toughness and its role in sport and performance. J. Sport Psychol. Action 8, 146–156. doi: 10.1080/21520704.2017.1332607

[ref9] Cowden R. G. Meyer-Weitz A. (2022). Mental toughness and performance under pressure: a review of contemporary literature. Front. Psychol. 13:857234. doi: 10.3389/fpsyg.2022.857234, 35865690 PMC9295738

[ref10] Creswell J. D. (2017). Mindfulness interventions. Annu. Rev. Psychol. 68, 491–516. doi: 10.1146/annurev-psych-042716-051139, 27687118

[ref11] Duggan J. Byrne S. Smith M. (2024). Trait mindfulness and adaptive coping across populations: evidence from longitudinal studies. Mindfulness 15, 45–62. doi: 10.1007/s12671-024-02110-2

[ref12] Filimon A. Ionut D. Pavel M. (2021). Mental toughness, perceived stress, and coping strategies in elite climbers. J. Sports Sci. Med. 20, 245–253.

[ref13] Fletcher D. Sarkar M. (2012). A grounded theory of psychological resilience in Olympic champions. Psychol. Sport Exerc. 13, 669–678. doi: 10.1016/j.psychsport.2012.04.007

[ref14] Gardner F. L. Moore Z. E. (2007). The Psychology of Enhancing human Performance: The Mindfulness-Acceptance-Commitment (MAC) Approach. Berlin: Springer.

[ref15] Gucciardi D. F. Gordon S. (2011). Mental toughness in sport: developments in theory and research. Int. Rev. Sport Exerc. Psychol. 4, 103–123. doi: 10.1080/1750984X.2011.607064

[ref16] Gucciardi D. F. Gordon S. Dimmock J. A. (2017). Towards an understanding of mental toughness in Australian football. J. Appl. Sport Psychol. 29, 15–31. doi: 10.1080/10413200.2016.1193614

[ref17] Gucciardi D. F. Hanton S. Gordon S. Mallett C. J. Temby P. (2015). The concept of mental toughness: tests of dimensionality, nomological network, and traitness. J. Pers. 83, 26–44. doi: 10.1111/jopy.12079, 24428736

[ref18] Hanton S. Fletcher D. Coughlan G. (2005). Stress in elite sport performers: a comparative study of competitive and organizational stressors. J. Sports Sci. 23, 1129–1141. doi: 10.1080/02640410500131480, 16194989

[ref19] Hayes A. F. (2018). Introduction to Mediation, Moderation, and Conditional process Analysis: A Regression-based Approach. 2nd Edn New York: Guilford Press.

[ref20] Jones G. Hanton S. Connaughton D. (2007). A framework of mental toughness in the world’s best performers. Sport Psychol. 21, 243–264. doi: 10.1123/tsp.21.2.243

[ref21] Kabat-Zinn J. (2003). Mindfulness-based interventions in context: past, present, and future. Clin. Psychol. Sci. Pract. 10, 144–156. doi: 10.1093/clipsy/bpg016, 38267955

[ref22] Kaufman K. A. Glass C. R. Pineau T. R. (2009). Mindful sport performance enhancement: mental training for athletes and coaches. J. Clin. Sport Psychol. 3, 335–342. doi: 10.1123/jcsp.3.4.335 PMC6157919 30271521

[ref23] Keng S. L. Smoski M. J. Robins C. J. (2011). Effects of mindfulness on psychological health: a review of empirical studies. Clin. Psychol. Rev. 31, 1041–1056. doi: 10.1016/j.cpr.2011.04.006, 21802619 PMC3679190

[ref24] MacKenzie B. Jones A. (2020). Psychological demands and performance in sport climbing. Int. J. Sport Psychol. 51, 101–118. doi: 10.7352/IJSP2020.51.101

[ref25] Mellalieu S. D. Neil R. Hanton S. Fletcher D. (2009). Competition stress in sport performers: stressors experienced in the competition environment. J. Sports Sci. 27, 729–744. doi: 10.1080/02640410902889834, 19424897

[ref26] Mojtahedi S. Alipour A. Zare R. (2021). Relationship between mental toughness and stress in competitive athletes. Sport Exerc. Perform. Psychol. 10, 558–570. doi: 10.1037/spy0000270, 27371692

[ref27] Mojtahedi A. Alipour A. Zare M. (2021). Mental toughness and perceived stress: evidence from elite athletes. Int. J. Sport Exerc. Psychol. 19, 345–360.

[ref28] Nicholls A. R. Polman R. C. Levy A. R. Backhouse S. H. (2009). Mental toughness, optimism, and coping among athletes. Personal. Individ. Differ. 47, 541–546. doi: 10.1016/j.paid.2009.05.001, 38826717

[ref29] Noetel M. Ciarrochi J. Van Zanden B. Lonsdale C. (2019). Mindfulness and acceptance approaches to sport performance enhancement: a systematic review. Int. Rev. Sport Exerc. Psychol. 12, 139–175. doi: 10.1080/1750984X.2018.1441558

[ref30] Oguntuase A. Wrigley C. Maxwell J. (2022). Integrating mindfulness into performance enhancement programs: practical strategies. J. Sport Psychol. Action 13, 245–257.

[ref31] Pan L. Li X. Zhang Y. (2024). Mindfulness-based interventions for stress reduction: a meta-analysis. Psychol. Sport Exerc. 66:102479. doi: 10.1016/j.psychsport.2023.102479

[ref32] Preacher K. J. Hayes A. F. (2008). Asymptotic and resampling strategies for assessing and comparing indirect effects in multiple mediator models. Behav. Res. Methods 40, 879–891. doi: 10.3758/BRM.40.3.879, 18697684

[ref33] Rogowska A. M. (2024). Mindfulness and sport performance: effects on stress and coping in competitive athletes. J. Sports Sci. 42, 540–550.

[ref34] Sadooghiasl A. Jafari H. Rezaei M. (2022). Effects of mindfulness on perceived stress and well-being: a systematic review. Mindfulness 13, 1892–1906. doi: 10.1007/s12671-022-01902-1

[ref35] Sappington R. Longshore K. (2015). Mindfulness and performance: implications for sport psychology consultants. J. Sport Psychol. Action 6, 10–20. doi: 10.1080/21520704.2014.972170

[ref36] Sheard M. Golby J. van Wersch A. (2009). Progress toward construct validation of the sports mental toughness questionnaire (SMTQ). Eur. J. Psychol. Assess. 25, 186–193. doi: 10.1027/1015-5759.25.3.186

[ref37] Tanaka H. Sato M. Kimura T. (2023). Mobile mindfulness interventions for athletes: effectiveness and engagement. Front. Psychol. 14:1178901. doi: 10.3389/fpsyg.2023.1178901

[ref38] Turkington R. Smith B. Jones R. (2023a). The protective role of mental toughness in athlete stress management. Front. Psychol. 14:1152. doi: 10.3389/fpsyg.2023.01152

[ref39] Turkington R. Smith J. Jones L. (2023b). Mental toughness and stress responses in high-performance athletes. J. Sports Psychol. 45, 112–128.

[ref40] Wister A. McPherson B. Ing R. (2022). Resilience and mental toughness: conceptual overlaps and implications for sport. J. Appl. Sport Psychol. 34, 75–90.

